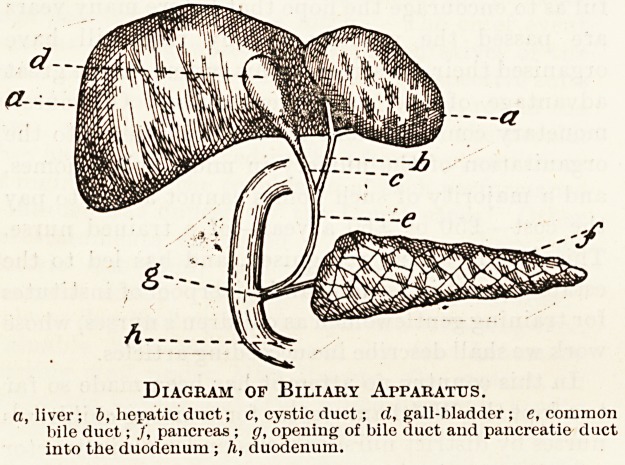# "The Hospital" Nursing Section

**Published:** 1906-07-28

**Authors:** 


### The Hospital.

### Nursing Section.

Contributions for "THE HOSPITAL," should be addressed to the EDITOR, "THE HOSPITAL" NURSING SECTION, 28 & 29 Southampton Street, Strand, London, W.C.

No. 1,036.—VOL. XL. SATURDAY, JULY 28, 1906.

### Notes on News from the Nursing World.

### THE QUEEN AND THE JUBILEE INSTITUTE.

THE sum of £2,000, which it was desired by the Queen should be raised and handed over to Queen Victoria's Jubilee Institute this year, has been secured, and Her Majesty has written to Lady Cadogan acknowledging the good work done by the ladies who have interested themselves in the matter. In her letter she asks that her thanks may be conveyed to the ladies of her Committee who have taken so much trouble to raise this sum. She esteems it very satisfactory that £300 in excess of the amount guaranteed should have been collected; and, having carefully considered the suggestion that 200 ladies should be asked to guarantee £10 per annum, she signifies her approval of it, adding that such a scheme ought to meet with great success, and should be the means of carrying on the undertaking in the future.

### HONOUR FOR MISS HUGHES.

AT a meeting of the Council of Queen Victoria's Jubilee Institute for Nurses held on Wednesday last week the President (the Master of St. Katharine's) reported that the Queen had accepted his resignation of the office of Chairman of the Council, which he had held since the foundation of the Institute, and the Hon. George Goschen was appointed Chairman, the Master of St. Katharine's retaining the position of President of the Institute. At the same meeting, and with the sanction of the Queen, the gold badge of the Institute was conferred on Miss Amy Hughes, the present general superintendent, in recognition of the valuable services she has rendered to the cause of district nursing. The decoration thus bestowed is thoroughly well merited, and we congratulate Miss Hughes upon being the possessor of a signal mark of distinction.

### NURSING IN INDIA AND LADY CURZON.

THE Queen has consented to become President of the Indian Nursing Association, and has contributed a donation of £100. The identification of Her Majesty with the undertaking launched by Lady Minto will certainly do much to ensure its success. While the wife of the present Viceroy must be congratulated upon the prompt support which she has already obtained for her Fund, the efforts of her predecessor at Simla in the same interests will not be forgotten. The late Lady Curzon, whose death has caused such widespread regret, was a warm friend of the movement for providing the sick and suffering in India with more trained nurses; and for this, among many reasons, she will always be remembered with gratitude.

### WHY THE ARMY NURSING SERVICE IS ATTACKED.

WE print this week a letter which goes far to strengthen the conviction that the attacks that are made from time to time on Queen Alexandra's Imperial Military Nursing Service are largely, if not entirely, due to jealousy. Our correspondent, "J. M.," evidently an orderly, or the spokesman of one, cannot endure the idea that women should be placed over men, "many of whom," he says, "are doctors in all but diplomas." Therefore he endeavours to discredit and impugn the capacity of the sisters under whom the orderlies have to work, congratulating himself that he has proved that "neither intelligence nor kindness can be the reason for giving the sisters such unreasonable—nay, unjust—powers over deserving, highly respectable men." His communication will help to clear the air.

### MR. HALDANE ON THE MOBILISATION OF NURSES.

THERE was one remark made by the Secretary of State for War at the distribution of prizes to the students and probationers at the London Hospital the other day which confirms the suspicions that Mr. Haldane fully recognises the important part which nurses would play in the unfortunate event of a great war. In responding to the vote of thanks which was accorded him, he said that his presence was not quite disinterested, for when he looked at the nurses before him the word "mobilisation" came to his lips. He added that "the London Hospital seemed likely to be a tower of strength to the Army if a moment of national emergency arose"; and we assure him with confidence that the London is only one of many great hospitals on which the Army may rely for help in the hour of need. It is for the War Office to initiate and propose any scheme for the mobilisation of nurses; the material is always ready.

### ENROLMENT IN TIME OF WAR.

AT the last meeting of the Infirmary Committee of the Portsmouth Guardians the medical superintendent submitted a report relating to the enrolment to an honorary nursing division in time of war of the nurses holding a certificate of training at the Poor-law Infirmary. The report stated that under the general regulations of the St. John Ambulance Association and Nursing Brigade, women holding certificates from recognised hospitals and institutions might be enrolled for service in war time. "Therefore," the report added, "applications had been made, and the nursing staff at the Infirmary had been enrolled as a nursing division, this being the first and only occasion on which a nursing division had been formed of nurses trained and in course of training." The report was adopted, but we do not see how the nurses of Portsmouth Poor-law Infirmary can become war nurses without without the assent of the War Office. Meanwhile, it would be interesting to learn what arrangements are contemplated by the Infirmary in the event of the nursing staff being called up.

### PLEASING INCIDENT AT THE LONDON TEMPERANCE HOSPITAL.

At the meeting of the Board of Management of the London Temperance Hospital on Monday a most interesting presentation was made by the matron on behalf of the nurses. On the morning following her return from her summer vacation the matron found upon her table a purse containing £130 and a card with the inscription, "To matron, the enclosed £130 for the new out-patient department, from the present and a few past nurses of the London Temperance Hospital, in token of their affection for their training school." The members of the Board expressed themselves highly gratified and impressed by this practical and kindly token of sympathy and interest by the nursing staff, and adopted a special resolution in order to record the event on the minutes of proceedings. The significance of the act is as noteworthy as its generosity.

### POOR-LAW SUPERANNUATION.

The question of the possibility of officials in the employ of the Local Government Board who contracted out of the Superannuation Act of 1896 being placed in such a position as would enable them to pay contributions for, and become entitled to, superannuation allowance has been raised by an assistant matron, who informs us that as the result of inquiries at headquarters she is still in doubt. As we are authoritatively assured, however, the case is that, having contracted out of the Act of 1896, under the provisions of Section 15 of that Act, and not under those of the Poor-law Officers' Superannuation Amendment Act of 1897, her decision is binding in all subsequent employment; but that under the Act of 1864 it will be open to the local authority to grant her a superannuation allowance when she retires. Of course, such an allowance would require the sanction of the Local Government Board, but this, we understand, would not be withheld in the event of it being certified that the duties during the period of service had been satisfactorily performed. It appears to us that the position is not what it ought to be, and that more elasticity is badly needed. But the Local Government Board can obviously only administer the law according to its legal interpretation.

### AN AMERICAN NURSE AND HER PATIENT.

A Brooklyn man received in May last a very extensive burn, and it was found exceedingly difficult to get the large wound to heal. The medical man in attendance found it necessary to graft on as many as 414 pieces of skin, of which 30 portions were contributed by the patient's nurse, Miss Snyder. Amongst those who also allowed skin to be taken from them were the patient's little daughter of twelve, his father, aged sixty, his wife, his doctor, and numerous friends. The result is stated to have been entirely satisfactory. An interesting experience of a male nurse in a case of this kind will be found on another page.

### CUBICLES IN THE BASEMENT.

The account which is given on another page of nursing at the Royal Hospital for Incurables shows that the accommodation for the staff at the esablishment on Putney Heath still leaves much to be desired. The sisters, who are fully trained, are well cared for in this respect, but the assistant nurses have to sleep in cubicles, many of them in the basement; and we surmise from the statement of the matron that they have not long enjoyed the privilege of such necessary furniture as a wardrobe for their own use. In their sitting-room they have neither lounge-chairs nor cosy corners, the inference being that the authorities are afraid of making them too comfortable. We think that this is bad policy, seeing that as a rule such institutions as the Royal Hospital for Incurables experience considerable difficulty in securing a full complement of suitable nurses, while the general hospitals and infirmaries whose matrons receive more applications than they can entertain, provide for each of their nurses a separate bedroom and sitting-room furnished on home lines.

### ROYAL VISIT TO WHITECHAPEL INFIRMARY.

On Tuesday, Princess Louise Augusta of Schleswig-Holstein paid a visit to the Vallance Road Infirmary, Whitechapel. In the absence of the matron, Her Royal Highness was received by Miss Taylor, assistant matron, who presented the Princess with a bouquet on behalf of the nursing staff. In the wards the Princess spoke sympathetically to many of the patients. To an elderly woman who is just recovering from a severe illness she took one of the choicest roses from her bouquet and handed it to her with bright and cheering words. She also manifested a keen interest in the children. She seemed greatly pleased with her visit, and expressed opinions highly complimentary to the institution.

### EXAMINATIONS IN AUSTRALIA.

The Central Board Examinations took place on Wednesday and Thursday, June 13 and 14. Fifty-two candidates presented themselves in Melbourne, eleven in Ballarat, eight in Bendigo, and one at Beechworth. Miss Burleigh, matron of the Melbourne Hospital, being in England, Miss Player, matron of the Children's Hospital, took her place. Miss Glover attended on behalf of the Council at the Ballarat sub-centre; Miss Mann, matron of the Women's Hospital, went to Bendigo; and Miss Düster, matron of the Wangaratta Hospital, went to Beechworth.

### THE ANNUAL OUTING TO GUY'S NURSES.

The entire nursing staff of Guy's Hospital was entertained by the Treasurer, Mr. Cosmo Bonsor, and his wife on Thursday and Friday last week at their residence, Kingswood Warren. They were conveyed by a special train each day, and, on reaching Kingswood station they found carriages waiting for them. Some, however, preferred to walk through the park. Refreshments were served on their arrival, and again before their departure. Various kinds of amusements were provided during the afternoon, and an excellent string band played at intervals. In acknowledging the vote of thanks which was proposed to the host and hostess for their kindness, Mr. Bonsor said that it always gave his wife and himself much pleasure to meet their guests from Guy's and they hoped to continue the outing each year.

### GARDEN FETE AT STAMFORD INFIRMARY.

IN aid of the funds of the Stamford, Rutland, and General Infirmary a garden fête was held in the grounds of that institution on Tuesday, July 17. There was a large attendance, and, thanks to the lady who superintended the work of organisation and by whom the idea was originated, the proceedings passed off without a hitch. The chief event was the "living whist," the representatives of the cards marching at the outset in their respective suits from the principal door of the Infirmary, round the lawn, accompanied by the players and the herald. Two hands were played in the afternoon, and three at night, and at the conclusion of each performance a minuet was danced by the Court cards. Other entertainments embraced a shooting gallery, an electric fish-pond, and the performance of a dramatic sketch. During the afternoon "Buller," a clever dog, took up £1 14s. 1½d. in a collecting-box. Valuable help was rendered by the Infirmary nursing staff under Miss Browne, the matron, in purveying flowers and fruit which had been kindly contributed by a number of friends. In the evening a programme of sports was gone through satisfactorily, the arrangements being admirably carried out by the Committee.

### A STRAWBERRY TEA.

THE nurses of the Sunderland Infirmary were entertained on Wednesday last week by their matron, Sister Mary, at an *al fresco* strawberry tea, which was given in the grounds of the institution to celebrate the thirty-third anniversary of her appointment to the office. In a short speech Sister Mary encouraged the junior members of the staff by expressing a hope that in their work just begun they might find the same happiness which had followed her throughout her labours. Her remarks were received by the hearers with acclamation.

### THE OFFICES OF THE PENSION FUND.

As some of our readers are under the impression that the offices of the Royal National Pension Fund have either already been removed, or will shortly be removed, from 28 Finsbury Pavement to Buckingham Street, Strand, we may state that the new buildings will not be ready for occupation until, at the earliest, quite the end of next year. The process of the demolition of the present buildings has not yet been commenced.

### NURSES AND TENNIS.

AT a meeting of the Manchester Education Committee last week exception was taken by one of the Council to the proposal of the managers of Swinton House School to provide a tennis-ground for the use of the nurses. Swinton House School is an institution for crippled children, and it was stated that there was no question of making a lawn, but only a little asphalted area, which is so situated that it can also be used as a playground for the children in bad weather. The Education Committee, ignoring the only objection offered, assented to the proposal, and the nurses at Swinton House will be able to enjoy their tennis. This is not the first occasion on which there have been murmurs against an institution providing nurses with means of recreation. No doubt it is possible to go too far in that direction, but a small outlay for a tennis-ground can hardly be condemned as an injustice to ratepayers, seeing that it tends to keep the staff in health and probably saves the cost of paying relief nurses and the expenses incidental to frequent changes.

### THE GARDEN AS AN ISOLATION HOSPITAL.

A CASE of malignant diphtheria lately occurred in a village in Oxfordshire, where there was no isolation hospital. The child died thirty-six hours after inspection. The father contracted the disease the same week. The house was small, and packed with children. The medical man ordered the patients to be taken into the garden and placed in a tent under the trees, where they were to live and sleep; in fact, to "camp out." The father was nursed out of doors the whole time, as an attempt to take him in at night resulted in his complaining of a feeling of suffocation. He is now convalescent, though not yet amongst his fellow-creatures. The tent was made with hayrick-sheetings, tied to apple-trees, shut at one end and left quite open at the other. After a few days the barn adjoining the house was converted into a bedroom for the rest of the children, the large doors being thrown quite back. The children had their throats sprayed every morning as a precaution. The mother nursed her child and husband entirely unaided until help could be procured; then a nurse took the patients in hand. He was, of course, completely shut off from the children. It is now four weeks since the father first showed signs of diphtheria, and no fresh case has occurred, either in the house or in the village. As may be imagined, this extemporised isolation hospital occasioned much talk and shrugging of shoulders amongst the inhabitants, with many prophesies of the terrible results which would follow such a rash act.

### THE LITTLE HOPPERS' HOSPITAL.

SINCE we referred, a fortnight ago, to the arrangements for the nursing of the sick during the hop-picking season, it has been found that the staff already engaged—a late sister and a late nurse at the London Hospital—might be usefully supplemented by a further appointment in connection with the Little Hoppers' Hospital at Five Oak Green, Kent. In the event of constant nursing not being required, it would be an advantage if the nurse could help among the pickers in the gardens.

### SHORT ITEMS.

ON Monday the Prince of Wales, as Grand Prior of the Order of St. John of Jerusalem, distributed at Marlborough House service medals awarded for conspicuous services to several members of nursing divisions of St. John Ambulance Brigade.

### The Nursing Outlook.

"From magnanimity, all fears above;
From nobler recompense, above applause,
Which owes to man's short outlook all its charm."

### TRAINING FOR CHILDREN'S NURSES.

### I. Some General Considerations.

The care of the child is becoming more and more a pressing question amongst civilised nations. In the past the lot of the eldest born member of an ordinary family has seldom been a happy one, because of the ignorance of its parents as to its management, and the experiments of which their ignorance has made the child a victim. This ignorance covers the selection of the first nurse, who may or may not have had any previous experience of a practical kind, and, as a consequence of the failure of the modern mother to suckle her own child, scurvy rickets is a disease which is confined to the children of the well-to-do and the very poor. The well-to-do rely too much on infant foods, and the very poor starve the children by giving them everything but the simple, natural food products which they need for their sustenance. In both cases the result is the same, and the constitution, if not the life, of the child is often threatened in consequence. In such circumstances a hearty welcome must be extended to every movement which aims at securing an adequate training for children's nurses.

We have been very much impressed with the necessity to encourage the employment of thoroughly qualified nurses in the care and training of young children, and are about to devote a column to this important department of national life. That department will include answers to questions affecting the care and management of children, and any subject which is calculated to secure the hygienic condition of the nursery, and the health and happiness of the children. Many children suffer because they are indisciplined or spoilt; the present is an age when there is far too little practical knowledge, and an absence of any real desire to attain it. Mothers are too apt to take the advice of all and sundry, and so make grievous mistakes. We heard of a mother recently who held, and practised, the theory that, as all the men told her, corporal punishment was good for children, she invariably instructed her nurses to slap them well as and when necessary. The effect on the children has been to make them rough and sullen. One lady describes them as being "little savages" in conduct. Such imbecility resulted probably in the children being often punished in this way without any adequate cause, and sometimes even unjustly. If a child has to be punished severely the punishment should be inflicted by the mother, or, in the case of boys, by the father, and any parents who delegate the infliction of corporal punishment upon their children to their servants exhibit a total absence of any real sense of responsibility, of the rights of the children, and of their duties as mothers and fathers. We are confident that any properly trained nurse who was told by a mother to freely slap the children would firmly insist that that was no proper part of her duty, and that she considered that kindness and the winning of the children's confidence were the right methods to adopt to secure discipline and the best results.

In the houses of the very rich it is now a common practice to place the nursery in the charge of a trained nurse, and the results have been so successful as to encourage the hope that before many years are passed the wealthy at any rate will have organised their nurseries on this system, to the great advantage of the rising generation. Of course, monetary considerations necessarily enter into the organisation of the nursery in middle-class homes, and a majority of such homes cannot afford to pay the cost—£50 or £60 a year—of a trained nurse. This fact is widely recognised, and has led to the establishment in London and Liverpool of institutes for training gentlewomen as children's nurses, whose work we shall describe in succeeding articles.

In this country no attempt has been made so far to adopt the Waltham system for training children's nurses by district nursing visits under an instructor who is a graduate nurse. In district nursing the students are divided into squads, in order that each in turn may have practice in every branch of the work. The visits are paid to lying-in mothers and their babies; at each home the mother is bathed, her hair is brushed and braided; the bed is changed; her room is put in order; her gruels and broths made; and her baby washed and dressed. Observations of her condition are recorded on slips, which the student takes to the doctor for his inspection and orders. At the earlier visits the work is done by the instructor herself, and then the student proceeds to do first one, and then another part of the work, under the instructor's supervision. Ultimately the student visits alone, and while so engaged is visited and criticised by the instructor. In order to make the work a success it is essential to obtain the co-operation of the practitioners who are in attendance on the mothers, and many of them should be invited to serve as instructors in the training school. In large cities, where the children of the poor are cared for by several associations, it ought not to be difficult for children's nurse-training associations to secure their co-operation, with a view to affording opportunities for their students to take charge of the babies in crêches and other establishments of the kind during the period they may be left there by the mothers.

### Abdominal Surgery.

By Harold Burrows, M.B., F.R.C.S., Assistant Surgeon to the Seamen's Hospital, Greenwich, and to the Bolingbroke Hospital, Wandsworth Common.

### AFFECTIONS OF THE LIVER AND GALL-BLADDER.

### Anatomy.

The liver and biliary apparatus are situated in the right upper part of the abdomen—epigastric and right hypochondriac regions—and are for the most part beneath the ribs and rib cartilages. The bulk of the liver lies immediately against the diaphragm, which, with the pleura, is all that intervenes between the liver and the right pleural cavity.

Diagram of Biliary Apparatus.

*a*, liver; *b*, hepatic duct; *c*, cystic duct; *d*, gall-bladder; *e*, common bile duct; *f*, pancreas; *g*, opening of bile duct and pancreatic duct into the duodenum; *h*, duodenum.

Bile after it has been secreted by the liver passes along the hepatic duct, then along the cystic duct and into the gall-bladder. From the gall-bladder the bile passes back again along the cystic duct into the common bile duct, and thence into the duodenum. The duct of the pancreas opens into the common bile duct just before the latter enters the duodenum, or the two ducts enter the duodenum by separate but adjacent orifices.

The object of mentioning these anatomical details will manifest when the affections of the liver and biliary apparatus are under consideration.

### Pathology.

Surgical affections of the liver and biliary apparatus may be due to (1) injury, (2) growths, (3) infection. So far as operative treatment is concerned the first two of these classes do not supply a large number of cases. Rupture of the liver is frequently associated with other severe injuries which may be quickly fatal. When rupture of the liver alone is present the symptoms are those of an abdominal injury with internal hæmorrhage, and this subject has been dealt with in a previous article. Mitigation of shock and preparation for immediate operation are the paramount indications.

Very rarely the gall-bladder may be ruptured. Cancer of the liver or bile ducts is not often amenable to surgical treatment.

So that there remains for discussion the subject of infections. The great majority of surgical diseases of the liver and biliary system are due to infection by micro-organisms. There are two main avenues by which these gain access. Firstly, they may spread from the duodenum up the bile ducts to the gall-bladder and liver. Secondly, they may be conveyed to the liver by the blood-stream. The liver is peculiarly liable to the latter mode of infection owing to the arrangement of the portal blood supply.

### Infection through the Blood-stream.

Briefly described, the portal system of blood-vessels is as follows: The capillary vessels of the intestines and other abdominal viscera open into veins which carry the blood to the portal vein. Now the portal vein, instead of leading the blood directly to the inferior vena cava and back to the heart, carries it instead to the liver, where the vein breaks up again into capillaries. So that any particle of septic matter entering the blood-stream from the abdominal organs is carried to the liver, in the capillary vessels of which it is extremely likely to become lodged.

So that suppuration in the liver is one of the complications for which it is necessary to keep on the look out in any case of abdominal sepsis, such as appendicitis, ulceration of the large intestine, and so forth.

### Infection from the Intestine.

The biliary system may be compared aptly to the appendix with regard to infection from the bowel. Just as the most frequent cause of appendicitis is infection from the bowel, so the chief cause of inflammation of the biliary apparatus is infection from the bowel. And the anatomical consequences of infection in the two cases are fairly comparable. In the case of the bile ducts it may lead to transient swelling of the mucous membrane of the ducts and temporary obstruction to the flow of bile, with jaundice; it may lead to the formation of gall-stones; and, if severe, may cause perforation or gangrene of the gall-bladder, with consequent peritonitis.

### Hydatid Disease.

There is one curious form of liver infection which can hardly be classed with the other forms. This is hydatid disease. It is uncommon in this part of the world, but is not rare in Australia and some other countries. Hydatid disease of the liver is caused by a parasite, which gains entry to the alimentary canal chiefly in contaminated drinking water. From the intestine the parasite migrates usually to the liver, sometimes to other organs. Having become established in the liver, it causes a large cyst or several cysts to form. In the course of time the parasite may die, in which event the cyst will shrivel up and probably cause no further trouble. But if the parasite does not die, the cyst, which is filled with clear fluid, will grow larger and larger, and in course of time, if untreated, may kill the patient by the pressure it exerts on important organs. Or before it reaches this size the hydatid may perforate the diaphragm and burst into the lung, or it may burst into the peritoneal cavity.

Sometimes suppuration occurs in the cyst, and leads to the rapid formation of a large abscess.

The treatment of hydatid disease is always surgical. In doubtful cases an exploring syringe is inserted into the liver; if this be done and clear fluid drawn off, care should be taken that the fluid obtained is not lost, because it will be necessary to have it examined. Hydatid fluid has certain characters, the presence of which will enable the surgeon to make a correct diagnosis.

### The Nurses' Clinic.

### THE DISTRICT NURSE AND ENTERIC FEVER.

As with a few precautions it is usually possible to avoid contracting or conveying the infection of enteric fever, the care of this complaint is frequently undertaken in district work. The great difficulty with regard to successful nursing is that for various reasons (the gradual nature of the attack, the position and often the dimness of the eruption, the semi-independent life of the majority of the patients, etc.) the early symptoms are commonly overlooked or mistaken, and the disease is far on in its course before doctor and nurse are summoned. One nurse told me that at her first visit she found the patient, a strong young man, sitting in a straight-backed chair and eating a dessert of nuts and apples. The doctor calculated that he was then in the seventh or eighth day of his illness, and he died shortly after.

The disease occurs all over the world and at all seasons of the year, but more especially in the autumn. Patients are usually between the ages of ten and thirty, but may be younger or very much older. I have known a mother and her grown-up son struck down at the same time. On the other hand, I have known seven young people sicken in one house, and their elders remain untouched.

Infection comes through exhalations from the patient's skin and lungs, or from his evacuations; the last are said to be more dangerous when decomposed than when fresh. The period of incubation is about three weeks, but is believed to be less if the disease is contracted by drinking tainted water.

Mild enteric fever lasts from seven to fourteen days, and often causes the patient so little inconvenience that there is difficulty in confining him to bed, or even to one room. Owing to this recklessness many originally slight cases have ended in death, in addition to spreading infection broadcast. The first need of every enteric fever case is absolute rest in bed. Statistics show that in the large majority of fatal cases this essential precaution had been neglected for six or seven days, or even longer.

In an ordinary case the patient's mind is clear throughout, and any delirium is a serious sign which should be reported at once to the doctor. If there is no relapse he will be convalescent at the end of a month, but the convalescence is probably a longer and more dangerous period than in any other form of disease. No attempts should be made for at least three months to return to the ordinary way of life. In severe enteric fever the temperature is higher (sometimes 105° F.), the weakness is more marked, there is great restlessness and inability to sleep.

The most dangerous and probable complication is peritonitis, which may occur in the third or fourth week. Even in mild and favourable cases the end of the third week is always an anxious time. Bronchitis (to some extent) generally occurs, and pneumonia (usually fatal) sometimes supervenes. In order to relieve the lungs modern practitioners do not insist on the patient lying continuously on his back, but allow him to be slightly tilted to one side or the other by an arrangement of pillows. This change of posture answers the double purpose of keeping off bedsores, always imminent where there is much weakness and emaciation.

The food of typhoid patients is not as exclusively confined to milk as it was formerly. Beaten up eggs and very milky tea are allowed; but the old rule holds good, "No food that would not readily pass through a fine sieve." It is impossible for the district nurse to be too emphatic or too frequent in her warnings as to diet, especially when the patient begins to get a little better, and the friends' fear and anxiety die down. I have known a patient who was progressing favourably thrown back for many weeks by eating a plum cake specially baked for him in a saucer by his devoted mother. During the nurse's absence she had suggested preparing this dainty; the lad was too weak to speak, but she saw the hungry gleam in his eye and the deed was done.

Owing to differences in constitution baths cannot safely be regarded as part of the ordinary routine, but must be regulated in each case by the doctor's explicit orders. The necessity of using a bed-pan must be explained to the patient's friends, and they must be shown how to do it with safety to themselves and others. They generally need to be warned that urine as well as fæces must be disinfected. The best disinfectant is izal 1-100, or carbolic 1-20.

When the temperature has been normal for seven days, fish, soup, and boiled chicken may be added to the diet, but they must be given in small quantities, and the effect carefully watched. About three pints of milk daily will be needed; in some cases it must be peptonised. If the patient suffers from thirst he may have small pieces of ice to suck, or cold water slightly flavoured with lemon. The doctor will give directions as to alcohol, and also with regard to aperients, enemas, etc.

The friends must be warned to keep their aprons and sleeves scrupulously clean, soaking them in disinfectant if they receive the smallest stain; to wash and disinfect their hands every time after attending to the patient; and to avoid inhaling his breath. The effluvium arising from the evacuations can be overcome by receiving them in disinfectant and covering the vessel immediately with a thick cloth, wrung out in disinfectant. All the patient's bed and body linen must be soaked in strong disinfectant. According to the supposed origin of the disease the nurse must give warnings with regard to drains, water, etc.

If peritonitis occurs it is as a rule accompanied by sudden and severe pain, and may be followed by death within from six to thirty-six hours. Occasionally it comes on slowly, there is less pain but considerable vomiting, and it frequently ends in death in the course of a few days, resulting either from exhaustion or perforation of the intestine.

If hæmorrhage appears during the first fortnight and is small in amount, the symptom is not considered of great importance, but should, of course, be reported to the doctor; but if blood is lost later on, and in large quantities, the patient is in great danger, and if the flow repeats itself two or three times the patient almost invariably dies.

A relapse sometimes sets in about the tenth or twelfth day

**THE NURSES' CLINIC—*Continued.***

after the temperature has become normal. The second attack, as it may be called, lasts from a few days to a fortnight, is rarely as severe as the original illness, and is seldom fatal. Second and third relapses are not unknown.

After very severe cases of enteric there is occasionally some mental weakness, and until health is entirely re-established serious study must be as carefully avoided as strenuous bodily exertion.

Just as in measles, the general character of the epidemic varies in severity, and this factor has probably more effect upon mortality than individual constitution, or slightly more or less favourable conditions of nursing attendance.

### Midwifery amongst the Poor.

Suffering humanity is all alike. Whether we are called to a mansion, a villa, or a slum, we have to take the same scrupulous care to avoid sepsis and ensure—humanly speaking—a quick return to health. All nurses know how very great their difficulties are in the courts and byways of great towns, but it must be admitted that inoculation is a strong factor in the case of the extreme poor and that they thrive and flourish in surroundings where the denizens of villadom would certainly succumb.

If a nurse has been warned beforehand that a birth is expected she would do well to visit the mother and give her a few hints on preparation for the event. I am supposing, of course, that she is a certified midwife and is to undertake the case alone. A relative or neighbour will usually assist at the time and afterwards give an eye to the children, cooking, and housekeeping. If the patient is a primapara she may be glad if the nurse would tell her if she has all the needed clothes, etc.; so often when it comes to washing the baby the binder or pins or needle and cotton are absent, and though these are small things that nurse may have in her bag, it is not a good plan to supply them as a matter of course, it will only be the thin edge of the wedge.

If it is possible—and I am aware it is often impossible—persuade the woman to have her bedroom scrubbed out and a supply of clean linen available. In cottages this might be done, but in the slums when one family one room is found it is practically out of the question, still cleanliness is a gospel which can always be preached, and who knows but what the seed may fall on good ground? As a rule the poor work up to the very day and almost hour of delivery, so advice about gentle, regular exercise would be superfluous in their case.

It is a great advantage to know beforehand your patient and her circumstances; for instance, if, as I have known a tin milk-can was the largest and only utensil available, arrangements might be made that some basins could be lent for the occasion. In summer time when fires are not likely to be burning it is a good plan to take a "Sirram" kettle and spirit stove with you, they are so handy and really invaluable when boiled water is required. The maternity bag, which every district nurse possesses, is re-examined and stocked after each case and the mackintosh well carbolised. For the benefit of those readers who have not yet qualified as midwives I venture to give some of the contents :—

A Higginson's syringe; a boiled glass vaginal nozzle wrapped in sterilised gauze; antiseptic tablets; small glass bottles of boric acid lotion, glycerine, and boracic; permanganate of potass crystals, tincture of ergot and eucalyptus ointment; absorbent wool; clean soft old linen; blunt pointed scissors; thread ready knotted in strands of three or four; nail-brush and soap; catheter preferably of glass ready boiled and wrapped in sterile gauze; thermometer; medicine glass; mackintosh, and the bag is furnished.

When the summons arrives start at once, complications, such as antepartum hæmorrhage may have arisen, and in any case delay is unjustifiable. On the way the friends can often give you the age of the patient and details as to previous labours and miscarriages, all of which must be entered in the register of cases. Arrived at the house, the first inquiry made is when labour began and if the waters have broken, and as a rule an enema saponis is given at once. This done, prepare the bed with the mackintosh you have brought to save the mattress and get the patient to put on her clean nightgown—which is afterwards pinned up round the waist out of harm's way—and petticoat and stockings. Notice if the pains are strong and frequent or whether they are failing. To prepare for making an examination scrub the hands and forearms for five minutes with soap and hot water and then steep them in 1-1000 corrosive sublimate. Then thoroughly wash the patient's external genitals with soap and water and swab with cotton-wool soaked in lotion, and again disinfect your own hands. Wait for a pain, then, raising the clothes with one hand, insert your lubricated, disinfected finger of the other hand straight into the vagina without touching any other part *en route*. Make as thorough an examination as possible, for if everything is normal it is very undesirable to examine again. Should you find anything abnormal requiring a doctor's assistance of course you send at once on the form provided for that purpose, otherwise let the woman walk about or sleep as she feels inclined. A cup of tea or gruel or some light food is acceptable at this time. Observe the duration of all the stages of labour to be entered later in the register. Do all you have been taught to prevent the rupture of the perinæum during the passage of the head and shoulders and notice carefully the presentation. Feel at once if the cord is round the neck and release it. Wipe the eyelids with boracic lotion as soon as the child is born and clear the mouth from mucus. From the moment the infant is born keep a hand firmly over the woman's uterus, and when the cord has finished pulsating get the helper to do so while you separate the baby and then resume the charge.

There is now a pause of from twenty to forty minutes when normally the placenta is expelled. It is received in the hand, the membranes gently twisted together and put on one side for examination. A small loss of blood generally follows, but the uterus should feel like a hard ball under the hand. The mother's parts are now cleansed with lotion, a clean warm pad applied, soiled articles removed from the bed, the binder if wished for put on, and the patient made comfortable. Some warm milk or an egg-flip should be given, the pulse counted and probably a sleep will follow.

Now the placenta is examined to see that it has come away entirely and take care that all soiled clothing is removed from the room. The patient is never left till an hour after the completion of the third stage and should be visited again within twelve hours, when the temperature and pulse are noted, and inquiries made about the lochia and bladder.

### To Nurses.

We invite contributions from any of our readers, and shall be glad to pay for "Notes on News from the Nursing World," "Incidents in a Nurse's Life," or for articles describing nursing experiences at home or abroad dealing with any nursing question from an original point of view, according to length. The minimum payment is 5s. Contributions on topical subjects are specially welcome. Notices of appointments, letters, entertainments, presentations, and deaths are not paid for, but we are always glad to receive them. All rejected manuscripts are returned in due course, and all payments for manuscripts used are made as early as possible after the beginning of each quarter.

### Nursing Incurables.

### INTERVIEW WITH THE MATRON OF THE ROYAL HOSPITAL FOR INCURABLES.

THE beauty of the surroundings of a hospital for ordinary surgical or medical cases is comparatively an unimportant point, because the patients will in a short time of necessity quit the establishment. But a hospital for incurables is the home of its inmates for life, and the natural charms of a position are appreciated in such circumstances. The Royal Hospital for Incurables stands within a short distance of Putney Heath, a handsome stone pile of buildings, approached by means of a large shrubbery, where noble trees and gay blossoms gratify the eye on every hand. As I entered the extensive grounds I met several of the patients being pushed, some by friends, others by nurses, in their invalid chairs, and learnt that a large number had been invited out to a garden tea in the neighbourhood.

"The nurses and patients seem to be on particularly happy terms," I remarked to the matron, Miss Stirling-Hamilton, as we chatted in her lofty sitting-room close to the large entrance hall.

"Yes, some of the nurses become much attached to those whom they have to tend, and who are so entirely dependent on their good offices, many not being able even to lift a finger for themselves."

### The Work of the Ordinary Nurse.

"How many does a nurse have under her charge?"

"She is responsible for five patients and for the tidiness and cleanliness of the ward. The actual scrubbing is done by wardmaids; but the work, of course, is not nearly so difficult as in an ordinary hospital. There is no washing and bed-making in the early hours of the morning. The nurses have their breakfast at seven, then serve the patients' at 7.30. Afterwards such as are able rise, with the nurse's assistance. The lift brings the first patients down into the general sitting-rooms (there are two for the women and one for the men) at ten, and goes on till 11.30, leaving the nurses to get their wards straight, which must be done by 12.30. The washing of some of the patients is a long process. Those who are badly crippled with rheumatism—and rheumatism in some form is responsible for the sad condition of two-thirds of our patients—need each finger, for instance, to be washed separately, and dried by passing a piece of lint between the fingers. The sufferers could not bear the friction of an ordinary towel. This little detail alone will show the difference between nursing an ordinary hospital patient and an incurable case. The prevention of bed-sores in those who have in some cases been bedridden for thirty or forty years needs much care and attention, though I am glad to say that we are very successful in this matter. At one time we had 212 patients in the house and not a single bed-sore, but it is impossible with certain patients to avoid them sometimes."

### The Training.

"I suppose you have to engage nurses who are especially physically strong, because of the lifting?"

"We find that short nurses are not advisable, but the lifting, even of very heavy patients, is more a question of knack than of actual muscular strength, and it is amazing sometimes to see a patient lifted with ease by a nurse half her weight."

"Are the nurses trained before they come here?"

"Not generally. The sisters, of whom there are six, called here divisional nurses, have all received three years' general training, but we have to train our own assistant nurses. This is done in practical work by the sisters in the ward, and by myself by means of lectures. We receive them from twenty to thirty years of age—older than that I find, except in very rare cases, is a mistake—give them £18 the first year, £20 the second, and £22 the third year, with indoor uniform. If they wish to wear outdoor uniform they provide it themselves."

"Do they engage to come to you for three years?"

"Oh, no. Although they do not get a testimonial unless they remain for a year, they may leave at any time by giving a month's notice. In consequence, we cannot avoid more changes than occur in ordinary hospitals, but those who desire to go in for general training afterwards are usually glad to stop."

### The Question of General Training.

"Have you found any difficulty in getting the nurses accepted as probationers at the general hospitals after they leave you?"

"If I can speak thoroughly well of them, not the slightest. Since I have been here, which is between three and four years, I have sent four to the London Hospital—my own training school—and St. Thomas's, the Middlesex, the Royal Free, and several infirmaries are now accepting them. The grounding they have received here helps rather than hinders their future career."

"How many nurses are there in this hospital?"

"About forty-four. Two of these are holiday nurses, and take the place of those who are absent, either for the half-day, which is given once a fortnight, the whole day off, which comes once in six weeks, or the fortnight's leave due at the end of a year. In the same way one sister is holiday sister. Here, of course, we can never diminish our number of patients at any time, and a sister or nurse cannot satisfactorily do double work. Therefore no other arrangement is possible."

### Sisters' Duties.

"What salaries do the sisters receive?"

"£30 to £35; they can work up to £45, and they have charge of forty-five beds. A duty in which I find they are generally unskilled when they come to me is that of carving the joints for the patients. Each sister carves for her own ward, and when dinner is in progress in the dining-rooms there are ten of us carving as quickly as we can, so that the meals may be served in a hot and appetising condition. The ward kitchen, of which there is one on each floor, is particularly useful in cold weather, as it has a good big oven as well as a rack where things can be kept hot. To feed our 215 patients and staff a good deal of food is needed. For instance, for breakfast 45 lbs. of fried bacon are required for the one meal alone."

### Accommodation for Staff.

"You have no Nurses' Home?"

"No; I much wish we had. The nurses and the domestic staff have to be housed either in the basement or on the top floor of the building. We cannot even give separate rooms, except to the sisters, and those are large and well furnished; but the Committee have done much to improve matters in general since my advent. Now the nurses are no longer classed with the servants, they have a wardrobe as well as a chest of drawers, with marble top to serve as a washing-stand. The rooms in the basement are capacious and light, the four beds standing in the four corners. At night curtains are drawn across the room in the shape of a cross, forming four cubicles. The rooms on the top floor accommodate six. A wall down the centre reaches to within a few feet of the ceiling, and curtains make each half-room into three cubicles. The night

NURSING INCURABLES—*continued.*

nurses—there is a permanent staff of five, with a trained superintendent—also sleep at the top of the house, their apartments having double doors."

"And the male attendants?"

"There are eight, and they are trained in their duties by the sister under whom they work. They are, of course, especially useful in lifting, but they often make very gentle nurses."

As I left the building the matron showed me the nurses' dining-room and sitting-room on the ground floor. The table in the former, which was ready for tea, looked bright with flowers and fairly comfortable, but the latter was singularly unhomelike. There was a piano, but neither lounge chairs nor cosy corners, and I did not wonder that it was empty, the tired nurses retiring in preference during their off duty time to rest upon their beds.

### Incidents in a Male Nurse's Life.

### AN AMERICAN PATIENT.

I was engaged some time ago as male nurse in a well-known hospital in America. Having hitherto had only two years' experience, I was naturally very pleased. My duties were not arduous, and in a week or two I had quite settled down, and was very comfortable. About three months after my appointment a patient was admitted suffering from severe burns caused by molten lead. A considerable part of his chest and back was badly burnt, and for some time the result was doubtful. As usual in such cases, his progress towards recovery was slow, and it seemed almost impossible to get the skin to unite.

Our Medical Superintendent was a young man, who was very nice alike to nurses and patients. If a nurse did not understand anything, or the case was specially interesting, he would readily answer any questions and explain the nature of the illness, etc. Such a man was naturally much esteemed, and we were all glad to please him. One day he was standing by my patient's bed and remarked that the only thing to make a satisfactory cure was to graft on healthy skin. After thinking the matter over a few minutes, I said that I would give him the necessary amount of skin if he would accept my offer. He inquired my reasons for volunteering and I told him. After discussing the pros and cons of the case he accepted my offer, and remarked that I should find it inconvenient for a time, as he would require rather a lot. The operation took place, and he removed about 5¼ inches of skin from my back and left arm. The grafting was a decided success. I naturally went off duty for a time, and was pleased to hear such a good account of my patient. However, I longed to return to my work, and when I was able to do so I found the man very grateful and he thanked me many times for what I had done, and was counting the days when he would be convalescent. He was to be discharged on a Thursday, and on the day preceding I was stooping down to put his bed tidy and pulling the counterpane down, when I faintly remember getting a blow in the neck. At the time it seemed to me as if it had been dealt with a sledge hammer, and so heavy was it that I fell over at once, and in falling caught my head on the bed castor. I was still half conscious, and suddenly a great weight came on my chest, and I remembered nothing more till I woke, thirty-six hours afterwards, in bed, with a nurse and the medical superintendent standing over me. I naturally wanted to know what was the matter. I had a horrible pain in the left side and my head ached terribly. Nurse said I must keep quiet and go to sleep, and I obeyed. Next day when the doctor came he told me what had happened, and that I was suffering from a broken collar-bone, five broken ribs, and a scalp wound, into which seven stitches had been put. No wonder I thought that my ribs and head ached! It appears that the patient, without the slightest warning, had developed homicidal mania, and, after knocking me down with a bottle, he stood up in bed and jumped on me! He was taken to a padded room immediately after this, and eventually removed to an asylum. Later, when making fair progress towards recovery, I got a chill, entirely through my own fault, pneumonia set in, and I ultimately had to stay in that bed for eleven weary months. I thought I should not recover, but owing to the splendid nursing and the attention of the medical staff I pulled through. Never shall I forget the little nurse who patiently watched over me for those weary months. I could not express on paper the gratitude that I feel. Even now, though 3,000 miles away, I send her The Hospital regularly. I gradually got stronger, but for long was a wreck of my former self. Had it been in a fair struggle I should have overpowered the man, for I was very strong, having taken three gold medals for good development, being 6 ft. 2 in. in height and weighing 15 stone, with a cast-iron constitution, which I have to partly thank for my recovery. But being taken unawares I, of course, had no chance. The patient died fifteen months later, never having recovered his mental equilibrium.

### Practical Hints.

**We welcome notes on practical points from nurses.**

### WARD ECONOMY.

There is no place where economy is more practised or more needed than in the hospital ward, no matter how wealthy the place may be. Some object upon which to spend money can always be readily found, and to spend it upon the most necessary and the most important things should be the aim of all who are in the position to decide how entrusted money can be best used. To make this policy effectual the small things of every-day use, which, because of their abundance and constant supply, are used sometimes indiscriminately, must be carefully guarded and methodically apportioned, so that the scope for expenditure may not be curtailed by unwise and unnecessary waste.

A few hints which can be tried, and will be found quite practicable, are given below.

Bandages are always a large item on the "dressings" bill. To avoid waste, have a bandage roller, so that those unsoiled and used for clean cases may be used a second or even a third time for the same patient. Where possible choose the bandage made of "domette," which washes beautifully, and can be used over and over again. Domette bandages are dearer to start with, but in the long run they will be found durable, and therefore cheaper. They are, however, more suitable for some dressings than others. In cases where poultices and dressings need constant renewing, do not use a fresh bandage each time, but fasten either in its place by means of a many-tailed bandage made of old linen or domette; the former is preferable in cases where there is infection or much discharge, as it may be promptly burnt on removing it. Tie with tapes or use safety-pins. Often in applying fomentations to the limbs it is quite unnecessary to employ a new bandage each time; if the limb rests on a splint, strips of old linen or broad bandages may be placed underneath, and then tied over the top of the fomentation. This is a good plan, because it saves labour and material, and, above all, the constant necessary lifting or moving of the limb where the applications must be frequent. The patient thus benefits tremendously by this device.

When the "Potts'" fracture has united and the patient begins to stroll about on crutches, the injured leg needs a sling or support for a little time. Often one sees a bandage supplying this need. Why not use black webbing about 2½ inches in width with a buckle for fastening, thus enabling it to be used in various lengths as required? This is infinitely tidier and much less expensive in the long run.

Lint is very often another source of waste. Until she is told, the unthinking junior probationer finds it extremely adaptable when she is cleaning and polishing here and there; but with the fact pointed out that it is wasteful, and that other and less expensive things will do as well, she will wage an equally effective warfare armed with a collection of odd bits—cuttings of wool and lint, etc., from stock dressings—placed within a piece of common grey gauze, forming a sort of "dumpling." This is a splendid way of using up otherwise useless scraps.

When ice-bags are in vogue, instead of covering them with the usual pieces of lint before applying to the patient's skin, make little bags of flannel domette, which will wash, and can be used over and over again; besides which the flannel is a more suitable covering, both from the point of view of comfort of the patient and of preservation of the ice. In dressings where gauze is used for a "drain," instead of cutting one from the larger portions of the prepared aseptic dressings and wasting the rest, it is a good plan to keep a small jar of little pieces of gauze ready cut; this saves both labour and material again.

Where it is necessary to use splints for a lengthy period always have them protected by gutta-percha tissue or jaconette, so that they may be washed over with some antiseptic fluid and then rebandaged. In this way the padding keeps perfectly clean and dry.

To keep ice-bags in good condition, when not in use fill them with tepid water once in ten days or a fortnight for a short time. This keeps the rubber soft and pliable, and it is not so liable to crack. The expense of rubber goods ought to make one careful to preserve them. Admirable little articles for washing patients may be made by knitting coarse soft knitting-cotton into about an 8-inch square. In a women's ward their manufacture will be greeted with pleasure, for most patients who are well enough love to be busy over a little needlework or knitting. If desirable to use them again, they can be sterilized and cleansed by boiling with a little soda; besides this latter advantage the material is cheap, and they are easily made.

If soap of the long bar kind is used for washing the patients, it will be found to go much farther when ordered in large quantities and cut into pieces of the required size, which should be put into a linen bag and hung up to dry before use.

### Presentations.

East Riding Asylum, Beverley.—Miss Macalister, matron of the East Riding Asylum, has been presented, on the occasion of her marriage to Dr. Mervyn A. Archdale, first assistant medical officer, with a silver tea service and oak tray from the nurses and staff

Perth Hospital.—Miss Annie Anderson, matron of Perth Hospital, Western Australia, has been presented, on her retirement after six years' service, with a silver trinket box and gold chain from the home staff, a gold bangle, a silver tray, brushes, and mirror from the resident nursing staff.

### Should Nurses be Registered?

(Reprinted from the "Lancet," July 21, 1906.)

The question of the nature and amount of the recognition to be extended to duly trained nurses is one which has greatly exercised the minds of many estimable people, both in and out of Parliament, and on it, there can be no doubt, different opinions may easily be supported by at least a semblance of argument. Medical men, for example, are known to be quite divided in opinion upon the matter. A large proportion of "fully trained" nurses maintain, and many of the matrons and other ladies who have been responsible for their training, maintain perhaps even more strongly that the instruction which they have received is of such a character as to deserve recognition and even protection from the State, in order that the public, as in the case of the medical profession, may at least be enabled to distinguish qualified from unqualified nurses when, if such be their pleasure, they may give their confidence only to the former. A registration by the State and a registration by the Royal British Nurses' Association have each had their advocates, advocates of whom it may, perhaps, be said that the so-called "claims" of the nurses have not been wholly free from a tendency to obscure, in their minds, the convenience and welfare of the institutions or classes by which nurses are chiefly employed. We do not in the least degree blame the champions in question for aiming at all that they can get, or even at a little more than is likely to be conceded to them; but our very tolerance of this position renders it the more necessary that the demands which have been put forth on behalf of nurses should be somewhat jealously scrutinised, and that the consequences likely to follow from granting them without reserve should be thoroughly understood by all who are concerned.

A very important difference between the sexes, which in this particular instance should certainly not be overlooked, arises from the fact that a man who becomes qualified as a member of any profession or calling usually depends upon it for his livelihood for the remainder of his days, and continues to practise it, with the necessary consequence that his knowledge is kept alive and in working order. A woman, on the other hand, is always liable to be diverted from any special occupation by marriage and by consequent claims of domestic and maternal duties, and it is evident that in a calling like nursing, which affords only a slender remuneration and makes close and constant demands upon the time, it would seldom be possible for a married woman to continue its practice. If every trained nurse were put upon a State register, which was supposed to afford proof of her fitness for the duties of her calling, it would inevitably happen that a certain, even a large, proportion of the persons so registered would soon abandon that occupation on account of the claims of marriage, and would thus, either temporarily or permanently, fall out of the ranks of the calling which they had embraced. A certain proportion of them would afterwards be compelled, by widowhood or by adverse circumstances, to seek to return to their original pursuit; and any kind of professional register which failed to distinguish between nurses who had continued in practice, whose knowledge had been kept up to the high-water mark by regular exercise, and nurses who had been effectively instructed at some remote period and had since had opportunities of forgetting whatever they had known, would certainly be a document calculated in some instances to mislead. On this ground alone, if on no other, we must agree with the "Memorandum" issued by the Central Hospital Council for London in its declaration that registration, as ordinarily understood, would fail to enable the public, when engaging the services of a nurse, to distinguish between one who was efficient and one who was not. It would, of course, fail still more completely in enabling the public to discover the presence or absence of the special qualities or the special experience which render a nurse peculiarly fit for the charge of certain classes of cases, as, for instance, for the care of children or of the aged. A medical man, in selecting a nurse for a patient, would still have to be guided either by his own previous experience of her work or else by the description of her character and capabilities which he received from the responsible official of the hospital, society, or other organisation with which she was connected, for this sort of information could not be supplied by any official register.

The "Memorandum" of the Central Hospital Council, to which we have referred above and which bears on behalf of the Council the signature of Sir Henry Harben as chairman, is distinguished by the common sense and practical shrewdness naturally to be expected from that strong man and capable organiser; and it forcibly calls attention to the effects which would follow if some of the more exaggerated claims which have been made on behalf of "trained" nurses were admitted by the Legislature. The "Memorandum" states that Sir Victor Horsley and Dr. Langley Browne, speaking for the British Medical Association before the Select Committee of the House of Commons, suggested that it should be a legal offence for any woman to engage in nursing who had not been *fully* trained, and it supplies arguments for disagreeing with the views thus expressed. For it points out that, if the law were as the representatives of the British Medical Association would have it, a very large number of nurses, many of whom are giving satisfaction to their employers and to the public, would be prevented from earning their livelihood. It points out, further, that, while the work of some imperfectly trained nurses is bad, this is not true of the greater number of them, while the inferiority of service often arises from causes quite unconnected with any lack of technical training. Again, it may often be of importance to the poor to be able to obtain the services of a nurse who is willing to accept a lower rate of pay than is demanded by the fully trained, a position that could not be considered under a registration scheme.

The authors of the "Memorandum" have not been content with merely negative criticism, and, after calling attention to the defects which would be inseparable from any system of registration and to the fallacy inherent in any comparison between the training of a nurse whose knowledge must often be secondary in importance to her personal character and the education of a medical practitioner, they proceed to suggestions for meeting the recommendations in the report of the Committee of the House of Commons. That is to say, they show how the employer of a nurse, whether medical man or layman, may satisfy himself as to her training, and they propose also to obviate the alleged difficulty which may arise from a nurse being sometimes unable, owing to a change of officials or other causes, to obtain a record of her training. For the attainment of these objects they recommend that an Official Directory of Nurses should be instituted and maintained by State authority, and that every nurse who had been trained at a training school for nurses not carried on for private gain should be entitled to an entry in this directory, showing her name, the places, dates, and periods of her training, the nature and dates of any certificates which she may have gained, and any hospital appointments which she has subsequently held. Removal of a name from the directory should be consequent upon death, upon conviction of any criminal offence, upon notice that the person concerned had ceased from nursing, or upon failure for a specified time to respond to official communications. The publication of such a directory would, it is maintained, accomplish all that is really necessary, either in the interests of the public or in those of nurses, and would avoid many objectionable complications. The directory would leave each training school at liberty to develop its teaching on the lines best suited to its circumstances. No system of compulsory examination, with its attendant difficulties and evils, would be instituted. The real employer of a nurse—that is to say, the medical practitioner—would be able to ascertain from the directory the bearing of her training upon her fitness for the case for which she was required, while the public could learn whether a nurse's statements as to her training were accurate, and also, in most cases, where to seek for further information if it were desired. No deceptive pledges of efficiency would be given by any public institution, and no woman with an aptitude for nursing would be hindered from affording to the sick such services as she was able to render and they were willing to accept, or from receiving such modest remuneration for her work as she might be in a position to command. The conditions thus indicated would seem to make for the greatest happiness of the greatest number, and, while they would leave the "trained" nurse in undisputed possession of the distinctions which she had earned, would place no artificial difficulties in the path of her humbler, but often not less useful or less willing, sister.

### The Nurses' Bookshelf.

The Midwife's Case Book. By Dr. C. St. Aubyn-Farrer. (London: E. and R. Garrould, Edgware Road, W. Price 9d.)

This is a neat and compendious register of cases attended by a midwife, and in accordance with the requirements of the Midwives Central Board. The register and the midwifery temperature chart occupy two pages facing, so that the notes on the whole case can be viewed at once, including presentation, duration of first, second, and third stage of labour complications, condition of mother and child, and the temperature over a fortnight's attendance—divided into spaces for morning and evening as in a normal case. A red line across the chart indicates normal temperature, and another red line at 100.4°F. marks the temperature which, if it lasts for more than 24 hours, indicates to the midwife that medical aid should be obtained. Over and above this, each case is provided with a perforated sheet of paper for notes. The whole design coming from Dr. C. St. Aubyn-Farrer, Physician-Accoucher, and Lecturer on Midwifery at the Royal Maternity Charity of London, is just the thing that careful midwives desire to possess.

Advice to Women: On the Care of the Health Before, During, and After Confinement. With Hints on the Care of the Newborn Infant, and an Appendix on what to get ready for a Baby. By Florence Stacpoole. Fourth edition, enlarged. (London, Paris, New York, and Melbourne: Cassell and Company, Limited. 1906. Pp. 84. 1 illus. Price 1s. net.)

A fourth edition of this little work is a guarantee of its utility and practical common sense, while its appendix, with minute instructions "What to get ready for a Baby," ought to prove most useful to an expectant but inexperienced mother. Its conversational style and freedom from puzzling technical terms account in a great measure for its popularity among those who do not belong to the nursing profession, for whose help the book is chiefly intended. In the chapter on "The Care of the Newborn Infant," we think that some directions about the feeding of the child could with advantage have been added, whether the baby were breast or bottle fed. There are many prescriptions given in the book, mostly of a simple nature; but it is sailing rather near the wind to encourage the laity to drug themselves without medical advice. We notice in one of these prescriptions that tincture of opium is mentioned, a drug that in unqualified hands may lead to grave mischief. With these exceptions, the book is excellent.

### Everybody's Opinion.

ARE NURSES UNDERPAID?

"NURSE W." writes: "May I be allowed to say a few words respecting the correspondence going on in THE HOSPITAL, "Are Nurses Underpaid?" I can endorse what "Nil Desperandum" says, my own experience having been just the same, except that for over twenty years I have contributed about £15 a year towards the support of an invalid mother; my earnings as a private nurse never exceeding £30 a year, and for some years only £20 and £25 a year. I paid also £8 8s. into the Pension Fund, so that I had very little left either for clothes or holidays. I have, now that I am obliged to retire from work owing to my mother's illness and also because I have reached the age limit, a small pension of £15 a year from the institution where I worked, and also from the Pension Fund £9 10s. a year. I also live in the country, but a cottage at 2s. 6d. a week is quite out of the question, and I have to pay 5s. a week for a very tiny cottage. My amusements are walking and reading.

MENTAL NURSES.

"TWO TRAINED MENTAL NURSES" write: We wish to thank "Edinburgh Nurse" for her sentiments regarding "Mental Nurses." We, too, think that it is time the high standard necessary for the mental nurse of to-day should be recognised both by hospital nurses and by the public generally. Considering that the training for both male and female mental nurses covers a period of three years, surely they have a right to the title of "nurse" in preference to that of "ordinary nurse," or even the more doubtful "attendant." We had the misfortune to engage ourselves as mental nurses in a workhouse infirmary in the South of England recognised as a model training school, and our lives were most miserable owing to the insolence and arrogance of the raw probationer, who, in spite of the fact that we held charge nurses' positions, heaped on us all the petty indignities imaginable because, alas! we were not "trained" in their sense of the word. The fact that several of them were recruited from the ward-maid, domestic servant, and barmaid class may perhaps account for their ignorance. We hope that the day may soon dawn for the mental nurse to be given such full meed of praise and recognition as is justly her due for her self-denying, trying, but by no means uninteresting life.

"ARMY NURSING."

"J. M." writes: You are kind in expressing so high an opinion of Army sisters, but perhaps you will permit me to point out that they are not by any means all value and no waste. Sentiment and mistaken bigotry is their excuse for relief from certain work in garrison hospitals; and if the doctor or orderly was to do likewise, what then? Why, too, are these sisters placed on a different footing, and why do they act so differently to real nurses, with men appointed to act as lackeys in special or enteric wards, while they do a little decoration, fuss round, including also the praise-taking? Above all, what on earth good or just reason is there for placing them over men, many of whom are doctors in all but diploma? Lots of soldier patients are in favour of them, but what kind of men are they? Sisters pet and make favourites, of course, but with regard to who is the better nurse—the sister or the Royal Army Medical Corps orderly? The opinion of such men is of no value whatever. And as to who is the kinder, while severe orderlies are to be deplored, yet sisters do not overshoot the mark in their concern or kind consideration. It is thus seen that neither intelligence nor kindness can be the reason for giving the sisters such unreasonable, nay, unjust, powers over deserving, highly respectable men. And it is the opinion of many well able to give one, that nursing in the Army, as it is, with first women, and then men, all boxed up will never give proper results till either the one or the other be withdrawn. Or if this is not considered practical, then I do hope that the men will be allotted the mental and lock garrison work, while the sisters are reorganised as a real staff of active, and not honorary, nurses. For then, and perhaps not till then, will the public be able to see what value these sisters have been giving for the good money they have been receiving. Now, in conclusion, let me with much pleasure and fullness of heart add my testimony to several most perfect heroic women whom I have met, doing most nobly the part of real nurses. Women who never for a moment allow an orderly to do what they could do themselves, loved by the patient and respected by the Royal Army Medical Corps for their capable and kindly ways, and living honourable lives. And I only wish I could say this of the others.

### Appointments.

AYR DISTRICT ASYLUM.—Miss Mary Christie has been appointed assistant matron. She was trained at Aberdeen Royal Infirmary, where she has since been sister.

BRADFORD.—Miss Alice E. Hall has been appointed inspector of midwives. She was trained at the Clapham Maternity Hospital, and holds the certificates of the Central Midwives Board and the Royal Sanitary Institute. She has done private work in Coventry, has been charge nurse at the Union Infirmary, Wellington, Somerset, and has been midwife-in-charge of one of the district homes in connection with the Liverpool Lying-in Hospital.

BIRMINGHAM GENERAL HOSPITAL.—Miss E. W. Maclagan has been appointed massage and electrical sister. She was trained at the National Hospital, Queen Square, Bloomsbury, and at the South Devon and East Cornwall Hospital, Plymouth. She has since had charge of the massage and electrical department at the Derbyshire Royal Infirmary.

BRITISH HOME FOR INCURABLES, STREATHAM.—Miss Ellen Walker has been appointed staff nurse. She was trained at the Victoria Hospital for children at Chelsea and the Royal Hants County Hospital, Winchester. She has since been staff nurse and sister at University College Hospital, London.

CAMBERWELL WORKHOUSE, CONSTANCE ROAD, S.E.—Mrs. Hetty Dean Waters has been appointed staff nurse. She was trained at Croydon Poor-law Infirmary. She holds the certificate of the Central Midwives Board.

EPSOM UNION INFIRMARY.—Mrs. Annie Stredwick has been appointed charge nurse. She was trained at the Kingston Union Infirmary, where she has since been ward sister. She holds the certificate of the Central Midwives Board.

HOME FOR EPILEPTICS, MAGHULL, LIVERPOOL.—Miss Alice Hulme has been appointed assistant matron. She was trained at the Chester Infirmary, and has since been senior sister at St. Mary's Hospital, Manchester.

NORTH-EASTERN HOSPITAL, TOTTENHAM.—Miss Mildred M. Dampney and Miss Marie L. Wolfe have been appointed charge nurses. They were trained at the Fulham Infirmary, Hammersmith, W., and have since been doing private nursing at the Brooklyn Institute, Anerley, S.E.

ST. MARY'S HOME FOR INCURABLES, PARKSTONE, DORSET.—Miss Florence Woodbridge has been appointed staff nurse. She was trained at the Hastings and East Sussex Hospital, Hastings.

SOUTH EASTERN HOSPITAL, NEW CROSS.—Miss Beatrice Wilson has been appointed charge nurse. She was trained at Monsall Hospital, Manchester, and Wandsworth Infirmary, London.

### Novelties for Nurses.

(BY OUR SHOPPING CORRESPONDENT.)

THE "DUCHESS" BOOT POLISH.

NURSES who are contemplating going off for their holidays, and do not intend to waste unnecessary pennies upon boot-cleaning, cannot do better than procure a little brush, a piece of polishing cloth, and a tin of the "Duchess" boot polish. The outside of the box is decorated by a picture of the Duchess of Devonshire, and suggests something much daintier than material for "shining" foot wear, but the paste itself is excellent. Very little need be used, and it could not do its work better. The fact that it is made by Messrs. Stephenson Brothers, Limited, of Bradford—whose furniture polish is a household word—is a guarantee in itself.

### Notes and Queries.

**REGULATIONS.**

**The Editor is always willing to answer in this column, without any fee, all reasonable questions, as soon as possible.**

**But the following rules must be carefully observed.**

1. **Every communication must be accompanied by the name and address of the writer.**
2. **The question must always bear upon nursing, directly or indirectly.**

**If an answer is required by letter a fee of half-a-crown must be enclosed with the note containing the inquiry.**

*English Nurses in Rome.*

(201) Can you give me the names and addresses of any English nurses in Rome?—*Inspector of Midwives.*

There are English nurses at the Anglo-American Nursing Home, 265 Via Nomentana.

*Hop-pickers.*

(202) Are nurses needed for the hop-pickers this year, and to whom should I apply?—*Meads.*

See the notes on "The Hop-Picking Season" in our issues of July 14 and to-day.

*Paralysed.*

(203) Can you tell me of any home or institution that would admit my husband, who is paralysed? He can pay £1 per week.—*M. L. B.*

Write to the National Hospital for the Paralysed, Queen's Square, Bloomsbury, London, W.C. The Secretary may possibly be able to help you. If not, advertise.

*Midwifery.*

(204) Is there any institution where I can get free training in midwifery?—*Cheltonian.*

All the training institutions charge a fee. Sometimes, however, by advertising you can hear of a position where training is given for work done.

*Housekeeping.*

(205) Can I get a post as assistant housekeeper for three months in a hospital, just to gain experience? I have some hospital training, a first-class cookery diploma, and have had 2½ years' experience in a private family.—*King's Co.*

We do not think that any institution would care to receive any officer for three months. Nurses going for so short a time have to pay.

*Insurance against Accident.*

(206) I have seen a paragraph in The Hospital saying that people could insure against operations. Is this correct?—*Palatine.*

The Ocean Accident Company, 44 Moorgate Street, London, insures against illness, including operations.

*Consumption Sanatoria.*

(207) Can you give me the address of sanatoria where a poor woman suffering from consumption could be admitted?—*Queen's Nurse.*

You will find a list of sanatoria in "Medical Homes," price 6d., The Scientific Press, 28 Southampton Street, Strand. You do not state if early or late stage of illness, or we might have given you fuller details.

*St. John's Ambulance.*

(208) Can you tell me how to belong to the St. John's Ambulance Association?—*B. G.*

Write to the Secretary, St. John's Gate, Clerkenwell.

*Grey Hair.*

(209) Is there any cure for premature grey hair?—*Eastbourne.*

We fear that there is no cure, but the progress is sometimes arrested by improvement in health and the use of stimulating lotions.

*Children's Nurse.*

(210) Where can I be trained as a children's nurse?—*Campbell.*

At the Norland Institute, 10 Pembridge Square, London, W.; at St. Monica's Home, 28 Upper Tooting Road, S.W.; at the Home, Flora Villa, The Wrythe, Carshalton; or the Princess Christian College, Wilmslow Road, Manchester.

**Handbooks for Nurses.**

| | Post Free. |
|---|---|
| "A Handbook for Nurses." (Dr. J. K. Watson) ... | 5s. 4d. |
| "Nurses' Pronouncing Dictionary of Medical Terms" | 2s. 0d. |
| "Art of Massage." (Creighton Hale.) ... ... ... | 6s. 0d. |
| "Surgical Bandaging and Dressings." (Johnson Smith.) ... ... ... ... ... ... ... | 2s. 0d. |
| "Hints on Tropical Fevers." (Sister Pollard.) ... | 1s. 8d. |

Of all booksellers or of The Scientific Press, Limited, 28 & 29 Southampton Street, Strand, London, W.C.

### For Reading to the Sick.

SACRED SYMBOLS.

It was not then a poet's dream
. . . . . .
Which bids us see in heaven and earth,
In all fair things around,
Strong yearnings for a blest new birth
With sinless glories crowned.

*John Keble.*

Everything in the world is in some sort symbolical of some greater, truer existence of the higher world. Christ is the True Light, the True Vine; *i.e.* that which is symbolised in the outer world by the Light and the Vine. In taking symbolical titles to Himself, Christ is not merely adopting to Himself a name which has its primary meaning in some lower phenomenon of the outer world. On the contrary, He is giving us the spiritual key to nature, so that when we see the material object, our heart may recognise the spiritual object which it indicates.—*R. M. Benson.*

A Temple there has been upon earth, a spiritual Temple, made up of living stones, a Temple, so to say, composed of souls; a Temple with God for its light, and Christ for the High Priest, with wings of angels for its arches, with saints and teachers for its pillars, and with worshippers for its pavement; such a Temple has been on earth ever since the Gospel was first preached. This unseen, secret, mysterious, spiritual Temple exists everywhere throughout the Kingdom of Christ, in all places, as perfect in one place as if it were not in another. Wherever there is faith and love, this Temple is.—*Dr. Newman.*

The way to mount up is to go down. Every step we take downward makes us higher in the kingdom of heaven. Do you desire to be great? make yourself little. There is a mysterious connection between real advancement and self-abasements. God's instruments are poor and despised, they are busied about what the world thinks petty actions, and no one minds them. They are apparently set on no great works, nothing is seen to come of what they do; they seem to fail, they rise by falling. The more they abase themselves the more like they are to our Lord Himself, and the more like to Him the greater must be their power with Him.—*Dr. Newman.*

Hath He marks to lead me to Him,
If He be my Guide?
In His Hands and Feet are Wound-prints,
And His Side.

If I find Him, if I follow,
What His guerdon here?
Many a sorrow, many a labour,
Many a tear.

If I ask Him to receive me,
Will He say me nay?
Not till earth and not till heaven
Pass away.

*Rev. J. M. Neale.*

## Figures and Tables

**Figure f1:**